# Fluid and Bubble Flow Detach Adherent Cancer Cells to Form Spheroids on a Random Positioning Machine

**DOI:** 10.3390/cells12222665

**Published:** 2023-11-20

**Authors:** José Luis Cortés-Sánchez, Daniela Melnik, Viviann Sandt, Stefan Kahlert, Shannon Marchal, Ian R. D. Johnson, Marco Calvaruso, Christian Liemersdorf, Simon L. Wuest, Daniela Grimm, Marcus Krüger

**Affiliations:** 1 Department of Microgravity and Translational Regenerative Medicine, Otto-von-Guericke University, 39106 Magdeburg, Germany; jose.cortes@ovgu.de (J.L.C.-S.); daniela.melnik@med.ovgu.de (D.M.); viviann.sandt@med.ovgu.de (V.S.); shannon.marchal@med.ovgu.de (S.M.); daniela.grimm@med.ovgu.de (D.G.); 2 Research Group “Magdeburger Arbeitsgemeinschaft für Forschung unter Raumfahrt- und Schwerelosigkeitsbedingungen” (MARS), Otto-von-Guericke University, 39106 Magdeburg, Germany; 3 Institute of Anatomy, University Hospital Magdeburg, 39120 Magdeburg, Germany; stefan.kahlert@med.ovgu.de; 4 Research in Space Environments Group, UniSA Clinical and Health Sciences, University of South Australia, Adelaide, SA 5000, Australia; ian.johnson@unisa.edu.au; 5 Institute of Bioimaging and Molecular Physiology, National Research Council (IBFM-CNR), 90015 Cefalù, Italy; marco.calvaruso@ibfm.cnr.it; 6 Department of Gravitational Biology, Institute of Aerospace Medicine, German Aerospace Center, 51147 Cologne, Germany; christian.liemersdorf@dlr.de; 7 Institute of Medical Engineering, Lucerne University of Applied Sciences and Arts, 6052 Hergiswil, Switzerland; simon.wueest@hslu.ch; 8 Department of Biomedicine, Aarhus University, 8000 Aarhus C, Denmark

**Keywords:** rotating bioreactor, simulated microgravity, cancer cell, shear stress, cell detachment, in vitro metastasis

## Abstract

In preparing space and microgravity experiments, the utilization of ground-based facilities is common for initial experiments and feasibility studies. One approach to simulating microgravity conditions on Earth is to employ a random positioning machine (RPM) as a rotary bioreactor. Combined with a suitable low-mass model system, such as cell cultures, these devices simulating microgravity have been shown to produce results similar to those obtained in a space experiment under real microgravity conditions. One of these effects observed under real and simulated microgravity is the formation of spheroids from 2D adherent cancer cell cultures. Since real microgravity cannot be generated in a laboratory on Earth, we aimed to determine which forces lead to the detachment of individual FTC-133 thyroid cancer cells and the formation of tumor spheroids during culture with exposure to random positioning modes. To this end, we subdivided the RPM motion into different static and dynamic orientations of cell culture flasks. We focused on the molecular activation of the mechanosignaling pathways previously associated with spheroid formation in microgravity. Our results suggest that RPM-induced spheroid formation is a two-step process. First, the cells need to be detached, induced by the cell culture flask’s rotation and the subsequent fluid flow, as well as the presence of air bubbles. Once the cells are detached and in suspension, random positioning prevents sedimentation, allowing 3D aggregates to form. In a comparative shear stress experiment using defined fluid flow paradigms, transcriptional responses were triggered comparable to exposure of FTC-133 cells to the RPM. In summary, the RPM serves as a simulator of microgravity by randomizing the impact of Earth’s gravity vector especially for suspension (i.e., detached) cells. Simultaneously, it simulates physiological shear forces on the adherent cell layer. The RPM thus offers a unique combination of environmental conditions for in vitro cancer research.

## 1. Introduction

**Figure 1:**
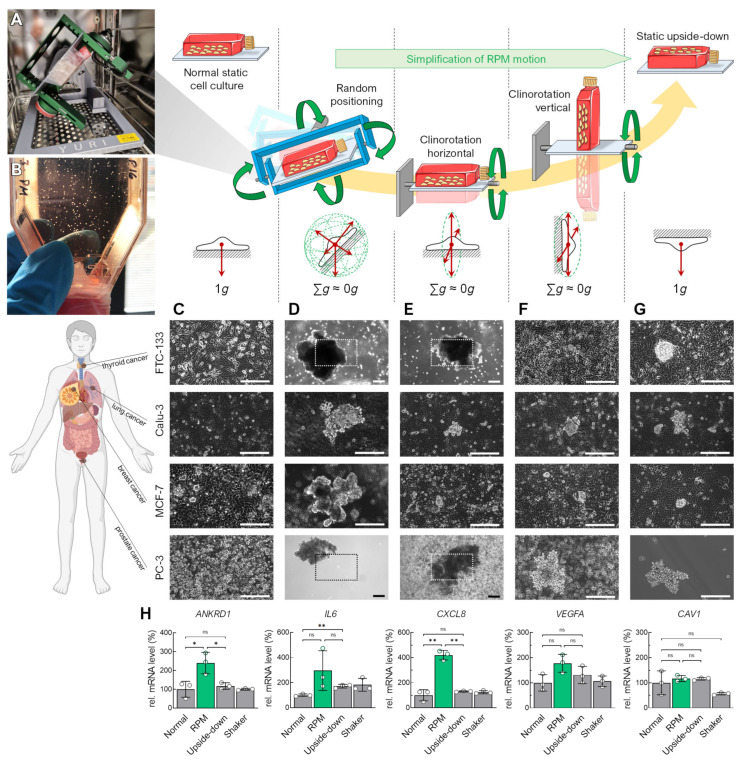
Spheroid formation of various adherent human carcinoma cell lines differently oriented with respect to Earth’s gravity vector for 72 h. Rotational movements of the cell culture flasks are indicated with green arrows. (**A**) Benchtop RPM in an incubator during an experiment with a T25 flask attached. (**B**) Visible spheroids formed after 3 days of random positioning in a cell culture flask. (**C**) Standard static cell culture. (**D**) Random positioning in real random mode. (**E**) RPM clinorotation mode with horizontal orientation of the T25 flask. (**F**) RPM clinorotation mode with vertical orientation of the T25 flask. (**G**) Upside-down static cell culture. (**H**) RNA expression changes in specific genes in FTC-133 cells that have been described previously to play a role in spheroid formation on the RPM. The plots show the mean ± SD ΔΔC_T_ values of three cell cultures performed in triplicate together with the individual data points. Scale bars: 300 µm. The outlined areas reflect the magnification of the field of view in most images except for larger spheroids formed in FTC-133 and PC-3 cells in RPM and horizontal clinorotation mode. * Independent sample *t*-test *p* ≤ 0.05, ** *p* ≤ 0.01, ^ns^ non-significant. Parts of the figure were drawn by using pictures from Biorender.com and from Servier Medical Art.

One of the most exciting aspects of biomedical research using microgravity provided by real microgravity platforms, such as International Space Station (ISS), is the formation of three-dimensional spheroids or organoids that seem to be enhanced under microgravity conditions lacking sedimentation [1,2,3,4]. These 3D cell cultures are more relevant to an organism’s complex biology than traditional two-dimensional monocultures of cells and provide a potential means to minimize animal experiments. Cancer cells exposed to microgravity may also provide models for metastasis events [5,6]. Whilst orbital labs such as the ISS provide the gold standard for microgravity research, the logistics and extensive costs prohibit easy access to real microgravity on orbital platforms. Even unmanned orbital (satellites) or sub-orbital platforms, such as sounding rocket flights, are very rare and extremely costly approaches. Further gravity research platforms like parabolic flights or drop towers cannot provide sufficient time under microgravity conditions to address questions regarding, e.g., developmental processes. Therefore, rotating bioreactors such as 2D clinostats, rotating wall vessels, or the random positioning machine (RPM, Figure 1A) have become essential tools to provide simulated microgravity environments for cellular biomedical research on Earth [7]. The rotational motion of the fluid environment allows suspension cells to experience simulated free-fall conditions avoiding sedimentation over longer durations. For some cell systems, therefore, these microgravity simulations can provide data comparable to experiments conducted on orbital platforms in real microgravity. Interestingly, exposure of certain types of adherent cells to simulated microgravity induces characteristic spheroid formation in some cell types (Figure 1B). However, it is not evident what induces the detachment of cells from the growth surface and their aggregation toward spheroid formation. The genomic, transcriptomic, and proteomic data even on a single cell type are heterogeneous across various platforms simulating microgravity or the respective culturing conditions [8,9]. Previously, it was argued that the effects of microgravity on individual cells are primarily indirect, and that the mass of a single cell is too low to directly sense changes in the gravity vector [10]. Instead, forces acting from the outside, such as the hydrostatic pressure of the medium or the local microenvironment, should change how the cell responds to the microgravity environment. The RPM induces biologically relevant shear forces [11,12,13,14] that affect human cancer cells in adherent cell layers. Suspension cancer cells in the same culture experience lower shear and are therefore affected differently. Currently, the studies on the RPM-based cellular behavior of human cancer-derived cell lines are not fully concise. Therefore, a generalized model of RPM-induced metastasis does not exist at the moment. In the current study, we aimed to understand which influences lead to cell detachment and spheroid formation under different modes of action on an RPM. The outcome might aid in the identification of metastasis traits at the onset of and in the course of cancer spheroid formation.

## 2. Materials and Methods

### 2.1. Cell Lines and Cell Culture

The human follicular thyroid carcinoma FTC-133 cell line (passages 8–18) was purchased from Sigma-Aldrich (St. Louis, MO, USA). The MCF-7 mammary carcinoma cells (passages 5–9) and PC-3 prostate carcinoma cells (passages 36–40) were purchased from ATCC (Manassas, VA, USA). The Calu-3 lung carcinoma cell line (passage 19) was a kind gift from Prof. Heike Walles, the University of Magdeburg. The FTC-133, MCF-7, and PC-3 cells were cultured in the RPMI 1640 medium (Life Technologies, Carlsbad, CA, USA) supplemented with 10% fetal calf serum (FCS; Sigma-Aldrich, St. Louis, MO, USA) and 1% penicillin/streptomycin (Life Technologies) at 37 °C and 5% CO_2_. The Calu-3 cells were cultured in the DMEM/F12 medium + 2.5 mM L-glutamine + 29 mM sodium bicarbonate (Life Technologies) supplemented with 10% FCS and 1% penicillin/streptomycin. The cells were routinely checked using visual inspection and maintained at a maximum confluence of 90% for up to 2 to 4 days before passaging. At 24 h before each experiment, a cell density of 1 × 10^6^ cells (40,000 cells/cm^2^) per flask was seeded in uncoated T25 cell culture flasks (Sarstedt, Nümbrecht, Germany) to allow cells to adhere. For immunofluorescence staining, uncoated glass coverslips (Carl Roth, Karlsruhe, Germany) were fixed using sterilized vaseline (Edeka, Hamburg, Germany) in a T25 flask before seeding a cell density of 1 × 10^6^. For the cell cultures in channel slides, 20,000 cells/cm^2^ (microscopy) or 40,000 cells/cm^2^ (qPCR) were seeded in a µ-Slide I Luer Glass Bottom 0.8 (ibidi, Gräfelfing, Germany).

### 2.2. Rotating Cell Cultures

For the experiments (4 h, 24 h, 72 h), a desktop RPM 2.0 (Yuri, Meckenbeuren, Germany) was used in a HERAcell CO_2_ incubator (Thermo Scientific) at 37 °C, 5% CO_2_ without humidification. The medium was not specially pretreated. For random positioning, the RPM was operated in real random mode (two frames) at three different speed ranges: average speed 25°/s (range: 21–30°/s), average speed 60°/s (range: 50–70°/s), and average speed 90°/s (range: 80–100°/s). The RPM was operated in one frame mode for clinorotation with a constant speed of 60°/s. Before starting the rotation, the cell culture flasks were filled completely with medium, avoiding bubbles (any remaining bubbles were carefully removed using a pipette tip before closing the culture flask). Static controls were placed next to the RPM in the same incubator at the same environmental conditions.

### 2.3. Orbital Shaker Cell Cultures (FTC-133)

For the shaker experiments (72 h) with the FTC-133 cells, a Stuart SSM1 Orbital Shaker (Cole-Parmer, Vernon Hills, IL, USA) was used in a HERAcell CO_2_ incubator at 37 °C, 5% CO_2_ without humidification. The shaker was operated using fully filled T25 flasks at a speed of 60 rpm.

### 2.4. Flow Channel Cell Cultures (FTC-133)

An ISM827 Reglo peristaltic pump system (Ismatec, Grevenbroich, Germany) was used for the experiments (4 h, 24 h). The system consisted of a medium reservoir containing 70 mL of cell culture medium. Carbogen (5% CO_2_ in O_2_; Air Liquide, Düsseldorf, Germany) was continuously injected into the reservoir to supply CO_2_ and O_2_ to the cell culture. On top of the flask, a syringe attached to an empty flask served as a foam trap for the sterile filter of the exhaust gas. The medium was pumped in a closed loop from a reservoir via a channel slide with defined dimensions (50 × 5 × 0.8 mm) (ibidi) at a rate of 0.5 or 1 mL/min creating a calculated shear stress of 12.5 and 25 mPa [15]. To mimic the shear stress induced by bubbles, medium supplemented with defined bubbles was pumped via channel slides. A second peristaltic pump connected was added to the inlet tubing upstream the slide via a Y-adaptor. In this setup, one bubble was pumped every second.

The effect of an oscillating fluid flow was investigated in an oscillatory flow configuration ibidi pump system (ibidi), in which one cycle consisted of a change in flow direction every 7.5 s and a flow rate of 1 mL/min (corresponding to 25 mPa of shear stress [15]). A brightfield image of the same slide area was acquired for live visualization every 5 min.

### 2.5. Serum Starvation and Pharmaceutical Treatment (FTC-133)

After 24 h of seeding, the FTC-133 cells were washed once using phosphate-buffered saline (PBS; Life Technologies), synchronized for 4 h in RPMI 1640 with 0.25% FCS and 1% penicillin/streptomycin, following cultivation with RPMI 1640 medium supplemented with 1 µM ethanol-soluble dexamethasone (Sigma-Aldrich) for 72 h, as described in [16]. The synchronization procedure was also used to reduce the amount of nutrients present before an experiment.

### 2.6. Phase Contrast Microscopy

The cells were routinely visually inspected and imaged using an Olympus CKX53 inverted microscope in phase contrast mode and a magnification of 10× (NA: 0.25) or 20× (NA: 0.4; Olympus, Tokyo, Japan). An Axiovert 200M microscope at brightfield mode at a magnification of 40× (NA: 0.6; Carl Zeiss, Oberkochen, Germany) was used for live visualization during the flow experiments in channel slides.

### 2.7. Cell Tracking and Migration Analysis (FTC-133)

For cell tracking, the Manual Tracking Plugin in Fiji v1.54f (ImageJ, imagej.net) was used. The exported file for tracking was processed using Chemotaxis and Migration Tool 2.0 (ibidi). For each condition, three independent FTC-133 cell cultures in the channel slides (see section Flow Channel Cell Cultures) were observed for 24 h. To determine the migratory ability of the cells, the average cell position at the end of the observation was determined for individual cells (*n* > 40 cells/condition), as well as the distance traveled in µm and the time to detachment in min.

### 2.8. Immunofluorescence Microscopy (FTC-133)

The cells were cultured as described above. For fixation, the cells were washed thrice using 0.1 M phosphate buffer (PB; Na_2_HPO_4_/NaH_2_PO_4_; Carl Roth), fixed for 15 min at 4 °C in 4% paraformaldehyde (PFA; Carl Roth), and then washed with PB for 3 × 15 min on a shaker. The cells were stored in 0.1 M PB at 4 °C for a maximum of one day before staining. The cell membrane was permeabilized using 0.2% Triton X-100 (Carl Roth) and washed thrice with 0.1 M PB. Non-specific binding sites were blocked using 3% bovine serum albumin (BSA, Carl Roth) in 0.1 M PB for 1 h at room temperature (RT). Subsequently, the cells were labeled with the primary antibodies diluted in 0.1 M PB containing 1% BSA (listed in Table S1) overnight at 4 °C. The next day, the cells were washed two times using 0.1 M PB. The cells were incubated with the secondary Alexa Fluor™ 488 (AF488)-conjugated (Invitrogen, Life Technologies) or Alexa Fluor™ 647 (AF647)-conjugated (Invitrogen, Life Technologies) antibodies (Table S1) at a dilution of 1:500 (MRTF-A) or 1:1000 at RT for 1 h. For F-actin staining, Alexa Fluor™ 568 Phalloidin (Invitrogen, Life Technologies) was added at a dilution of 1:400 for 1 h, and then the sample was washed thrice with 0.1 M PB. The cells were washed again three times using 0.1 M PB and mounted using Fluoroshield™ with DAPI (4′,6-diamidino-2-phenylindole) to stain the nuclear DNA (Sigma-Aldrich). The slides were examined using a ZEISS LSM 800 confocal laser scanning microscope (Carl Zeiss). To ensure comparability for intensity quantification, all the images were acquired with the same settings using the ZEISS Airyscan detector and ZEN 3.4 software (Carl Zeiss). The Airyscan processing settings were optimized for each antibody–wavelength combination and manually applied to the corresponding samples. To check for non-specific binding of the secondary antibody and thus a false negative signal, the secondary antibodies were applied to separate samples of the same condition without the primary antibody.

### 2.9. Immunofluorescence Analysis

To determine the localization of the transcription factors, we searched for the nuclear signal in the Z-position of the fluorescence image. The Z-position was used where the size of the nucleus was largest and the intensity of the nuclear signal was strongest. The cytoplasmic part of the signal was determined at the same Z-position. In this way, a correct measurement of the ratio between the nucleus and cytoplasm was ensured. Only 1 Z-position per file was used.

The resulting file was used in Fiji software v1.54f (ImageJ, imagej.net) to quantify the relative protein expression levels based on the sample’s fluorescence intensity using the Image Calculator tool. The relative intensities were measured according to the method of Shihan et al. [17]. To determine the ratio of protein localization between the nucleus and cytoplasm (N/C ratio), the mean fluorescence intensities of the protein of interest were measured for the nuclear area and the cytoplasm area. Using the Freehand Selection tool, the nuclear area was determined based on the DAPI signal. The total cell area was determined by selecting the background without cells and subtracting it from the total area. The cytoplasmic area was determined by subtracting the nuclear area from the cell area. The intensity measured in the cytoplasmic area is the average signal from the cytoplasms of all cells in this image. The signal intensity from the nuclear area was divided by the signal intensity of the cytoplasmic area. This corresponds to the individual N/C ratio for the respective image.

### 2.10. mRNA Isolation and Quantitative Real-Time PCR (FTC-133)

The procedure was performed in the same way as described in [18]. To obtain sufficient mRNA out of the cells grown in channel slides, a higher cell number of 40,000 cells/cm^2^ and a µ-Slide, 0.8 Luer, ibiTreat (ibidi) were used. The primer sequences used in the quantitative real-time PCR can be found in the Supplementary Materials (Table S2). The samples were measured in triplicate and evaluated by using the comparative threshold cycle (ΔΔC_T_) method. 18S rRNA was used as the housekeeper reference due to its stable expression in dynamic FTC-133 cell cultures [18].

### 2.11. Statistical Analysis

Statistical evaluation was performed using SPSS Statistics v29 (IBM, Armonk, NY, USA). The non-parametric Mann–Whitney U test was used to compare samples (biological replicates) from different culture conditions. For experiments in which only a few samples were available (<5 biological replicates), an independent sample *t*-test was used. All data are presented as mean ± standard deviation (SD). In most plots, the individual data points are shown additionally. The sample size for each experiment is provided in the figure legends.

## 3. Results

### 3.1. Alteration of the Gravity Vector

The operating principle of rotating microgravity simulators is based on a continuous change in the Earth’s gravitational vector direction on the cells. We aligned diverse human carcinoma cell lines (FTC-133, Calu-3, MCF-7, PC-3) in various positions with respect to the Earth’s gravitational vector field (Figure 1). In general, these cells grow as monolayers in standard (static) cell culture (Figure 1C). Although MCF-7 cells sometimes tend to form multiple cell layers, the cells remain adhered to their surface. Exposed to the RPM, the adherent cancer cells formed spheroids above the cell layer within 3 days (Figure 1D).

To determine which orientations or movements of the cell cultures contribute to spheroid formation during random positioning, and whether a particular gravity vector orientation is sufficient to form three-dimensional aggregates, we examined rotating cell cultures mounted horizontally (Figure 1E) or vertically (Figure 1F) on an RPM operated in 2D clinostat mode. After 3 days, the FTC-133 cells aligned parallel to the rotational axis formed spheroids, whereas the cells oriented perpendicular to the rotational axis remained adherent. Calu-3, MCF-7, and PC-3 cells formed smaller spheroids in the perpendicularly oriented rotation than in the other rotations (Figure 1F). Interestingly, we also observed the formation of spheroids from all cell lines in a static, inverted cell culture (Figure 1G). These occurred in low-density cultures, making three-dimensional growth due to overgrowth an unlikely cause. Here, we detected new cell growth on the opposite (lower) side of the culture flask, caused by cell detachment from the cell layer and sedimentation. Spheroid formation from an inverted vessel suggested that the cells must hang “upside down” for a time for spheroids to form. The spheroids were more prominent in the rotating cell cultures, especially on the RPM. Accordingly, simply inverting the cell culture flasks only partially replaced random positioning for spheroid formation. This is also reflected in the upregulation of certain typical genes (e.g., *ANKRD1*, *IL6*, *CXCL8*) in FTC-133 cells that have been described previously as involved in RPM-induced spheroid formation (Figure 1H) [19,20,21]. To verify whether better mixing of the culture medium on the RPM (60°/s) compared to the static cell cultures could explain these RPM-specific gene regulatory effects, an additional experiment was performed using normal-orientated cell cultures on a rocket shaker (60 rpm). The shaker had only a minor effect on *CAV1* expression and cannot explain the molecular effects found in the RPM cultures (Figure 1H).

**Figure 2:**
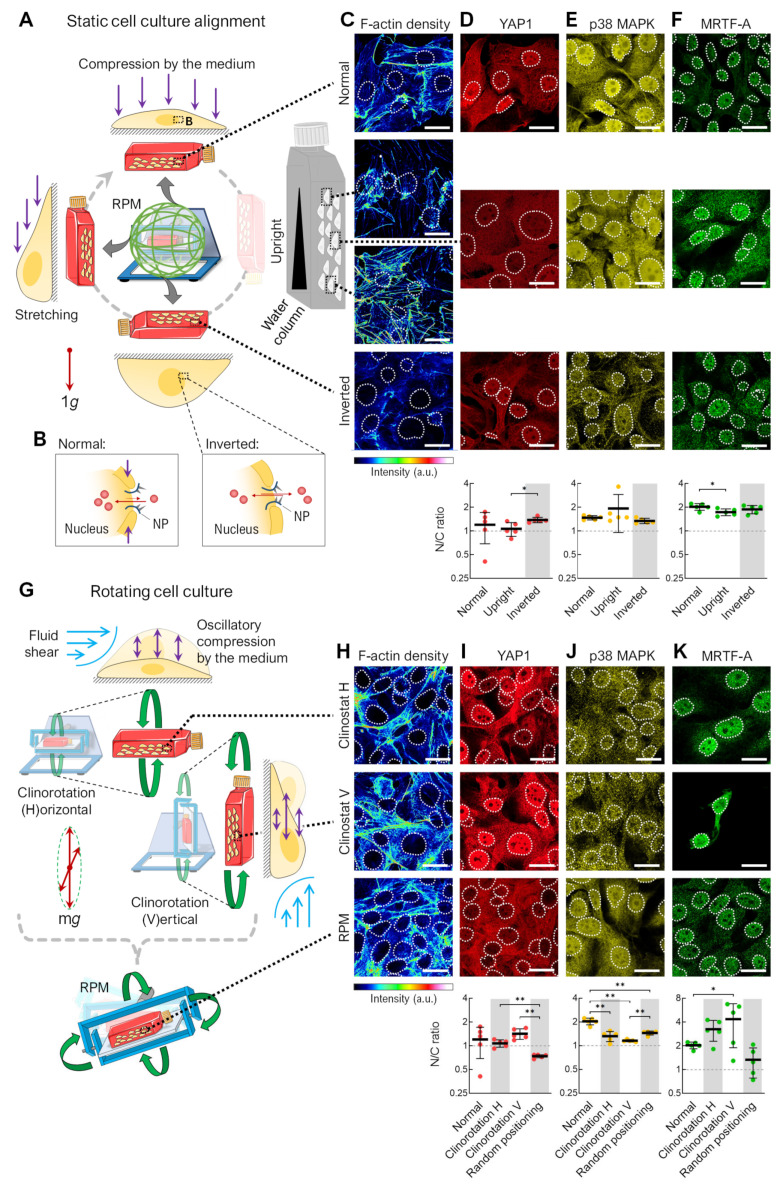
F-actin density and transcription factor localization in the remaining adherent cells in differently oriented and rotated cell culture flasks. (**A**) Static cell cultures in different orientations: normal cell culture, upright flasks, and inverted flasks. (**B**) Effect of cell compression on nuclear transport of transcription factors (red circles) via nuclear pores (NP). (**C**) F-actin density (phalloidin staining) and immunofluorescence of the mechanoresponsive transcription factors (**D**) YAP1, (**E**) p38 MAPK, and (**F**) MRTF-A after 72 h (*n* = 5 for each condition; one representative picture is shown). Outlines of the nuclei as indicated using DAPI staining (not shown) depicted as dashed lines. (**G**) Rotating cell cultures: horizontal clinorotation (60°/s), vertical clinorotation (60°/s), and random positioning (average speed 60°/s). (**H**) F-actin density. (**I**) YAP1, (**J**) p38 MAPK, and (**K**) MRTF-A localization after 72 h (*n* = 5 for each condition; one representative picture is shown). Outlines of the nuclei are shown as dashed lines. (Below) The relative mean nuclear–cytoplasmic (N/C) ratio of transcription factors was measured for at least 15 cells (5 pictures per condition). Static cell culture was set as reference. The experimental conditions where the spheroid formation was observed are shaded gray. Scale bars: 300 µm. The fluorescence images in this figure were optimized to visualize protein localization and unsuitable for comparative protein level quantification. * Mann–Whitney *p* ≤ 0.05, ** *p* ≤ 0.01. Parts of the figure were drawn by using pictures from Servier Medical Art.

Since the FTC-133 cell line showed the greatest differences in spheroid formation at different orientations and is a well-characterized cell culture model in altered gravity conditions [3,22,23], we used this cell line for further studies. Next, we imitated the different orientations of cell culture flasks occurring on the RPM with static and rotating cell cultures (Figure 2). The RPM positions the cell culture flask via rotation in all spatial directions (Figure 2A). Both the cells and the culture medium surrounding them are subjected to gravity. In a completely filled T25 cell culture flask (*h* = 24 mm), the cell layer experiences 240 Pa (*p* = *ρ* × *a* × *h*) of hydrostatic pressure at a normal orientation. This pressure compresses the cells and the nuclei, which can also promote the nuclear transport of transcription factors (Figure 2B) [24]. In an upright cell culture flask, there is a gradient in hydrostatic pressure increasing from top to bottom.

In an inverted cell culture, there is no hydrostatic pressure from the cell culture medium on the cells, making this orientation the most “force-free”. Cells in the inverted and at the top of the upright-oriented culture flasks showed lower F-actin filament density than in normal culture (Figure 2C). This could be related to the lower impact of mechanical forces lacking a large medium column, with corresponding hydrostatic pressure above the adherent cells. These results could support the idea that cells do not sense the gravity vector directly due to the low mass of their organelles but instead sense the indirect effects of gravity on larger structures, such as the surrounding culture medium [10]. There was minimal change in the nuclear or cytoplasmic distribution of the mechanosensitive transcription factors YAP1, p38 MAPK, or MRTF-A in the static cell cultures of different orientations (Figure 2D–F). These factors are important players in mechanosignaling, helping cells to recognize and respond to mechanical forces and adapt to changes in their microenvironment.

A cell culture on the RPM is not only subjected to different positions but also to different rotations, resulting in fluid shear forces and periodic compressions of the cell. These effects should be mimicked using two different types of 2D clinorotation produced by utilizing only one rotational axis of the RPM (Figure 2G). The F-actin density was similar in the clinorotation and RPM samples, indicated by some stress fibers (Figure 2H). A translocation to the cytoplasm was observed for YAP1 when the cells were exposed to random positioning (Figure 2I), and a statistically significant alteration in p38 MAPK subcellular localization when the cell culture flask was rotated with horizontal orientation (Figure 2J). We observed stark, but highly variable, differences in the nuclear/cytoplasmic fluorescence ratio for MRTF-A; compared to normal cell culture, more nuclear distribution was observed after clinorotation regardless of the flask orientation, and more cytoplasmic distribution was observed after random positioning (Figure 2K).

In summary, rotating cell cultures, including RPM cultures, have a stronger effect on transcription factor translocation than static cell cultures, further emphasizing the importance of flow shear on the RPM. However, it should be noted that in the cell cultures in which spheroids are formed (Figure 2, gray-shaded areas), there is no uniform nucleation of the transcription factors studied. This may indicate that these signaling pathways are not involved in spheroid formation or that the cells are already adapted to the culture conditions after 72 h.

**Figure 3:**
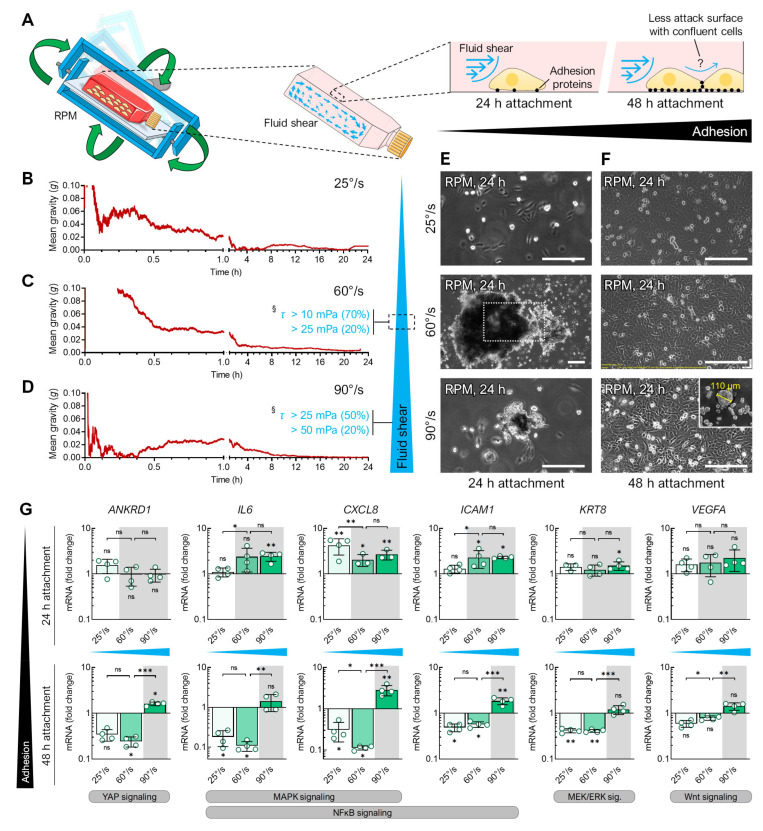
Spheroid formation of FTC-133 cells exposed to the RPM at different angular velocities for 24 h. (**A**) Illustration of fluid shear within cell culture flasks on the RPM. Time-averaged residual gravity levels and shear forces were calculated at RPM average velocities of (**B**) 25°/s, (**C**) 60°/s, and (**D**) 90°/s. Spheroid formation was examined in FTC-133 cultures that had previously been allowed to adhere to the bottom of the cell culture flask for (**E**) 24 h or (**F**) 48 h. The outlined area reflects the magnification of the field of view in most images except for larger spheroids compared at 60°/s after 24 h of normal cell growth followed by 24 h of exposure to the RPM. (**G**) Expression changes (compared to static cell culture) in gene transcripts known to be responsive to shear stress after 24 h exposure to the RPM operated with different velocities. The plots show the mean ± SD ΔΔC_T_ values of four cell cultures performed in triplicate together with the individual data points. The experimental conditions where spheroid formation was observed are shaded gray. Scale bars: 300 µm. * Independent sample *t*-test *p* ≤ 0.05, ** *p* ≤ 0.01, *** *p* ≤ 0.001, ^ns^ non-significant vs. static control. § Shear stress τ according to Wuest et al. [14]. Parts of the figure were drawn by using pictures from Servier Medical Art.

An RPM experiment with different angular velocities showed that the time-averaged cancellation of the gravity vector alone could not form spheroids (Figure 3). In microgravity experiments, the RPM typically operates at an average frame speed of 60°/s, resulting in fluid flow in the cell culture flask [14,25] (Figure 3A). In addition to the usually applied real random mode at a velocity of 60°/s, we performed experiments at velocities of ~25°/s and ~90°/s. All operating speeds resulted in a calculated time-averaged milligravity (~0.01 *g*) within a few hours (Figure 3B–D). Slower random positioning of the FTC-133 cells (average speed 25°/s) failed to induce cell detachment or spheroid formation (Figure 3E). Using higher RPM speeds (average speed 90°/s), much smaller spheroids formed compared to the typical angular velocity of 60°/s.

Assuming that a balance between cell confluency and fluid flow was needed to efficiently induce spheroid formation, we let the cells attach for 24 or 48 h prior to RPM exposure in all three angular velocities. Indeed, a longer period of cell attachment before RPM exposure affected the spheroid formation ability. The FTC-133 cells that adhered for 48 h did not form spheroids at any RPM speed (Figure 3F), except for one single small spheroid (110 µm) found at 90°/s (Figure 3F, insert). This effect of a prolonged adhesion phase before the start of the experiment was also observed in the PC-3 cells (not shown) and during the CellBox-1 mission, in which the cells became confluent due to a delay in the rocket launch and did not form spheroids during the experiment on the ISS [26]. We demonstrated that only under spheroid-forming conditions on the RPM was the expression of certain genes upregulated (e.g., *IL6*, *CXCL8*, *ICAM1*, *KRT8*) that have previously been described as targets of various mechanosignaling pathways (including p38 MAPK, NFκB, MEK/ERK) in human cells, among others (Figure 3G) [19,20]. Hence, both the fluid flow generated by the RPM and the adhesion force of the cells are important parameters for spheroid formation.

### 3.2. Fluid Shear

**Figure 4:**
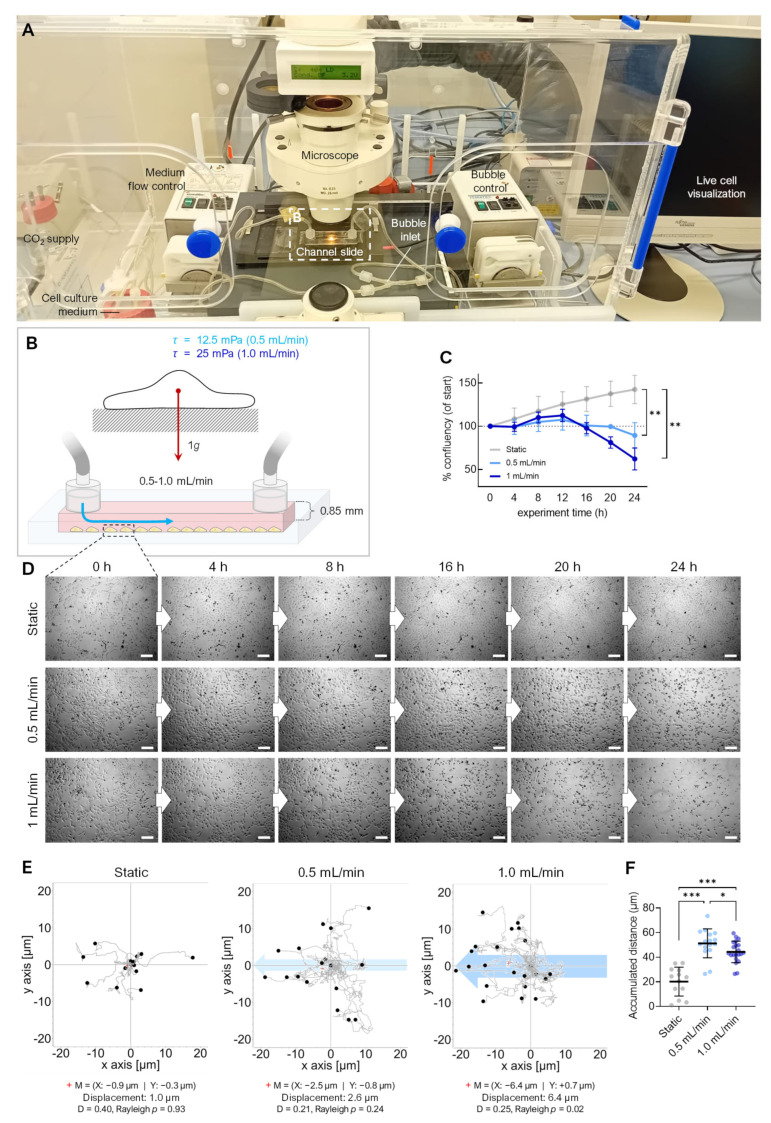
Effect of fluid flow on detachment of adherent FTC-133 cells. (**A**) Setup of the flow experiment. (**B**) Flow schematics. (**C**) Confluency of cell cultures over 24 h (*n* = 3). (**D**) Snapshots from the brightfield time-lapse recordings of one representative experiment with different flow speeds. (**E**) Migration traces of single cells in the flow channel perfused with different flow rates. The blue arrow indicates the direction of flow, and the position of cells (*n* > 40 cells for each condition) after 24 h is indicated by black circles. The red cross (+) indicates the mean value (M) of spatial cell migration (values are given below the plot). D: directness. (**F**) Cumulative migration distances of cells over 24 h at different flow rates. Scale bars: 300 µm. * Independent sample *t*-test (**C**) or Mann–Whitney (**F**) *p* ≤ 0.05, ** *p* ≤ 0.01, *** *p* ≤ 0.001.

Live-cell imaging on the RPM is not yet possible; therefore, we used a flow channel slide experiment to simulate the effects of RPM-induced fluid shear stress (Figure 4). Adherent FTC-133 cells were cultured in a channel slide connected to a peristaltic pump that perfused the medium through the channel at 0.5 and 1 mL/min speeds (Figure 4A,B). The fluid flow in the chosen dimensions of the channel slide (50 × 5 × 0.8 mm) produced shear stresses on the cells in the order of 12.5 and 25 mPa [15]. This stress was supposed to be similar to the fluid shear stress predicted to be observed on the walls of T25 cell culture flasks on the RPM at an average speed of 60°/s [14]. Both fluid flows significantly decreased the cell confluency within 20–24 h (confluency change at 0.5 mL/min: −11%, 1.0 mL/min: −38%) (Figure 4C). Time-lapse imaging showed cell detachment (Figure 4D, Video S1), migration, and rolling across the surface in the same direction as the media flow (Figure 4E). More cells detached at a flow rate of 1 mL/min, with initial cell detachment occurring after 12 h of flow exposure. This is consistent with prior observations of spheroid formation occurring after 16–24 h on the RPM [27,28,29]. Finally, the cells rounded off, detached, and were transported away by the flow. This observation over an extended period suggests that the detachment of the cell could be part of a biological process. In non-perfused cell cultures, no detachment was observed (Figure 4C), and the cell motility was significantly reduced (Figure 4F). Cell detachment is an essential step in RPM-induced spheroid formation; previous studies have shown that detached FTC-133 cells (i.e., suspension cultures) can form spheroids on the RPM. Interestingly, the proliferation of adherent cells was slightly higher at a flow rate of 1 mL/min compared with 0.5 mL/min, until approximately 12 h after the start of the experiment (Figure 4C,D).

### 3.3. Air Bubbles

**Figure 5:**
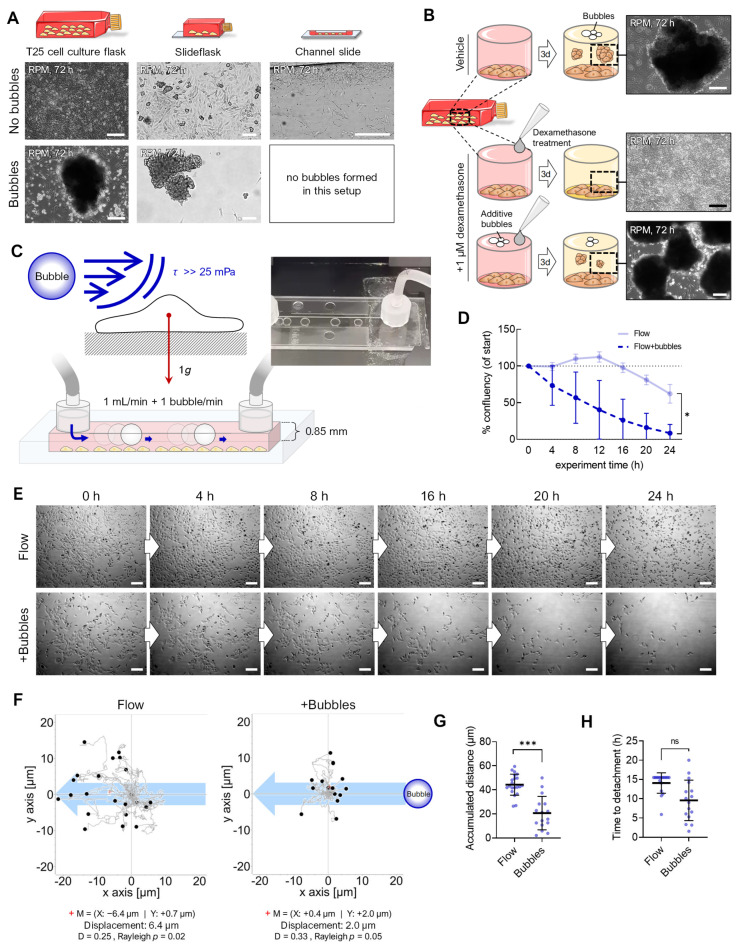
Enhancing effect of air bubbles on detachment of adherent FTC-133 cells. (**A**) Spheroid formation on the RPM depends on the culture flask geometry and the presence of air bubbles during the experiment. (**B**) Air bubbles were able to counteract the inhibitory effect of dexamethasone on the spheroid formation of FTC-133 cells on the RPM. (**C**) Setup of the bubble experiment. (**D**) Confluence of cell cultures over 24 h (*n* = 3). (**E**) Snapshots from the time-lapse recordings of one representative experiment with a flow rate of 1 mL/min without and with 1 bubble/min. (**F**) Migration traces of single cells in the flow channel. The blue arrow indicates the flow direction, and black circles indicate the position of cells after 24 h (*n* > 40 cells for each condition). The red cross (+) indicates the mean value (M) of spatial cell migration (values are given below the plot). D: directness. (**G**) Cumulative migration distances of cells over 24 h at a 1 mL/min flow rate with and without air bubbles. (**H**) Experiment time until initial cell detachment. Scale bars: 300 µm. * Independent sample *t*-test (**D**) or Mann–Whitney (**G**,**H**) *p* ≤ 0.05, *** *p* ≤ 0.001, ^ns^, not significant. Parts of the figure were drawn by using pictures from Servier Medical Art.

From previous RPM experiments, we knew that the observation of air bubbles at the end of an RPM experiment (including their size and number, compare Figure S1) influences the number of adherent cells remaining, depending on the cell line and flask configuration used (Figure 5A). In a recent study, we described that FTC-133 cells cultured on the RPM in the presence of 1 µM dexamethasone (to avoid cell clustering) did not show spheroid formation [16]. However, when additional air bubbles were added to the cell culture flasks containing an FTC-133 cell culture (bubble diameter: 0.5–8.3 mm at the end of the experiment), spheroid formation occurred on the RPM even during treatment with 1 µM dexamethasone (Figure 5B). The air bubbles in a cell culture flask on the RPM thus appeared to be able to detach even “stickier” cells. RPM experiments usually are initiated using completely filled cell culture flasks devoid of air bubbles using de-gassed culture media. Therefore, it is challenging to determine when and where bubbles originate from (Figure S1). They occur sporadically, even without cells, which indicates an incomplete de-gassing of the medium and/or, e.g., temperature fluctuations during the filling of the flasks. Furthermore, repeated biological or technical replicates (identical cells, flasks, medium, treatment, and cell number) resulted in differing numbers and sizes of bubbles present at the end of the RPM experiment. In various experiments, we found a correlation between the formation of spheroids and the presence of bubbles in culture flasks. The size and geometry of the culture vessel also played a role in RPM-induced spheroid formation. For example, we have not observed air bubble movements and spheroid formation of adherent cancer cells in channel slides. In a µ-well slide experiment, only one single bubble was formed that moved around the interior edges of the cuboid chamber and did not disturb the cells (Figure 5A).

Consequently, we modified the channel experiment, and air bubbles were pumped into the flowing medium at one bubble per second (bubble diameter: 5 mm; Figure 5C), using the same flow rate as in the shear flow experiments (1 mL/min). The cells detached at an earlier timepoint than without bubbles (Figure 5D,E; Video S2), suggesting bubble-enhanced cell detachment due to enhanced mechanical forces above 25 mPa (confluency change with bubbles: −92%, without bubbles: −38%). In addition, we observed significantly reduced cell migration (20.7 µm vs. 44.3 µm) when bubbles were present (Figure 5F,G). This could also be related to a faster detachment of the cells during migration (Figure 5H).

### 3.4. Flow Versus Random Positioning—Mechanobiology of Cell Detachment

**Figure 6:**
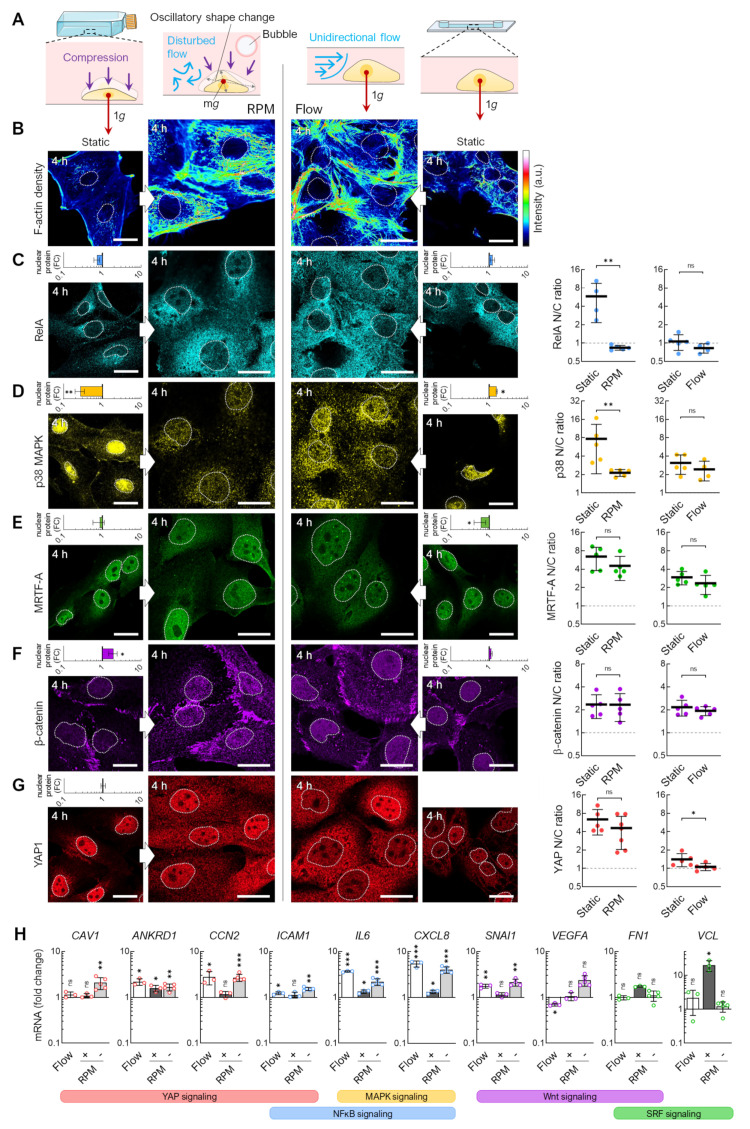
Transcription factor levels in random positioning compared to continuous flow. (**A**) Fluid motions acting on cells within different cell culture vessels. (**B**) Actin density and immunofluorescence of the mechanoresponsive transcription factors (**C**) RelA, (**D**) p38 MAPK, (**E**) MRTF-A, (**F**) β-catenin, and (**G**) YAP1 after 4 h. Outlines of the nuclei are shown as dashed lines. The small graphs indicate fold changes (FC) in nuclear protein levels compared to static cell cultures (*n* = 5 for each condition; one representative picture is shown). (Right) The mean nuclear/cytoplasmic (N/C) ratio of transcription factor localizations was measured for five overview images from independent experiments, each showing at least three cells. (**H**) Gene expression response after 4 h exposure to the RPM (60°/s) and continuous flow (1 mL/min). The plots show the mean ± SD ΔΔCt values of 3–5 independent experiments performed in triplicate. To mimic the channel slide’s low medium and nutrient volume, the RPM experiment was performed once identically to the flow (“+”, dark gray bars) experiment and once with a prior starvation phase (“−”, light gray bars). Scale bars: 300 µm. The fluorescence images in this figure were optimized to visualize protein localization and unsuitable for comparative protein level quantification. * Mann–Whitney (**G**) or independent sample *t*-test (**H**) *p* ≤ 0.05, ** *p* ≤ 0.01, *** *p* ≤ 0.001, ^ns^ non-significant vs. static control. Parts of the figure were drawn using pictures from Servier Medical Art.

After recognizing that fluid flow, as it occurs during the usual operation of the RPM in cell culture flasks, leads to the enhanced migration and detachment of adherent FTC-133 cells, we directly compared the mechanobiological responses of cells to flow- and RPM-induced shear forces (Figure 6). Because the number of cells in the channel slides decreased rapidly (compare Figure 5), we chose an examination time point 4 h after the start of the experiment. This also bypasses a possible adaptation of the cells that could be observed within 3 days. Shear forces might act differently on the same cell type depending on the culture vessel (T25 flasks and channel slides) and the corresponding possible exposure to fluid motions (Figure 6A). Nevertheless, in both culture conditions, we observed an increase in the F-actin density (Figure 6B) and altered localization (nuclear or cytoplasmic) of certain mechanosensitive transcription factors. Whilst both experimental approaches resulted in an altered nuclear protein content of RelA and p38 MAPK, the difference was only statistically significant for cells on the RPM (Figure 6C–E). Specifically, a decrease in nuclear p38 was observed on the RPM after 4 h, while an increase was observed in the flow channel (Figure 6D). MRTF-A nuclear localization was slightly decreased in the flow channel (Figure 6E). Subcellular localization of β-catenin remained unchanged. Conversely, the amount of β-catenin in the RPM cells was significantly increased (Figure 6F). Whilst there was no change in the nuclear levels of YAP1 on the RPM, YAP1 localization became more cytoplasmic in the cells under both exposure to RPM and flow (Figure 6G).

To investigate the mechanosignaling pathways, we investigated the mRNA expression of a panel of genes previously observed to be differentially expressed under simulated microgravity conditions [18]. The expression of these target genes was similar on the RPM and in the flow channel (Figure 6H). We found that *IL6*, *CXCL8*, and *ANKRD1* were upregulated in both RPM and flow conditions. We serum-starved T25 cell cultures for 4 h before starting the RPM. This procedure better mimicked the lower nutrient availability in the channel slides after the 24 h attachment phase and further approximated the gene expression changes to those of the cells from the flow channel experiment (Figure 6H, light grey bars). *CCN2*, *ICAM1*, *SNAI1*, *FN1*, and *VCL* showed similar regulatory behavior in both experimental setups.

**Figure 7:**
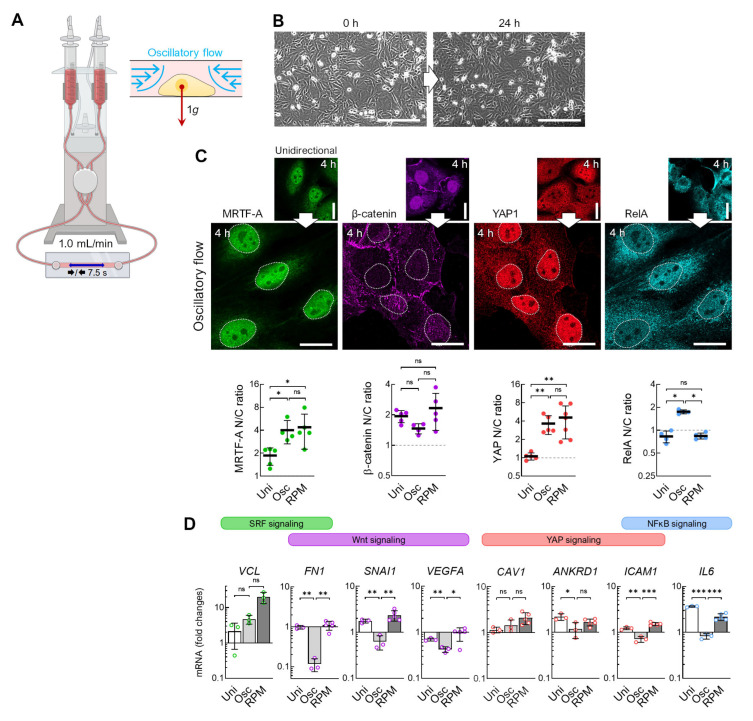
Transcription factor effects in response to oscillatory flow compared with continuous flow. (**A**) Setup for an oscillatory flow experiment (1 mL/min, direction change every 7.5 s). (**B**) Snapshots of cells in the channel before the start of the experiment and after 24 h of oscillating flow show reduced detachment of cells. (**C**) Nuclear transport of transcription factors after 4 h oscillatory flow (*n* = 5 for each condition; one representative picture is shown). (Below) Fold changes in nuclear protein levels compared to unidirectional flow. The mean nuclear/cytoplasmic (N/C) ratio of transcription factor localizations was measured for five overview images from independent experiments, each showing at least three cells. (**D**) Gene expression response after 4 h exposure to the oscillatory flow (Osc) compared to unidirectional flow (Uni) and to RPM culture (*n* = 3–5). Scale bars: 300 µm. The fluorescence images in this figure were optimized to visualize protein localization and unsuitable for comparative protein level quantification. * Mann–Whitney (**C**) or independent sample *t*-test (**D**) *p* ≤ 0.05, ** *p* ≤ 0.01, *** *p* ≤ 0.001, ^ns^ non-significant. Parts of the figure were drawn using pictures from Biorender.com (accessed on 30 October 2023).

The direction of the fluid flow in the RPM culture and the channel slide differs and changes continuously during random positioning. To test the effects of a discontinuous fluid flow on the cells, we utilized an oscillatory fluid flow of 1 mL/min and a flow direction change every 7.5 s (Figure 7A). Unlike a unidirectional flow, fewer cells detach when exposed to an oscillating flow (Figure 7B). In addition, β-catenin was significantly reduced in the nucleus after 4 h, whilst RelA, MRTF-A, and YAP1 were increasingly localized in the nucleus (Figure 7C). Thus, MRTF-A and YAP1 show a similar behavior as in cells exposed to the RPM, in contrast to β-catenin and RelA. qPCR confirmed that the SRF target gene *VCL* was upregulated, and the Wnt/β-catenin target genes *FN1*, *SNAI1*, and *VEGFA* were uniformly downregulated (Figure 7D).

In summary, FTC-133 cells cultured on the RPM and those cultured in a flow channel show similar behavior regarding actin condensation, the translocation of mechanoresponsive transcription factors, and the expression of the target genes of the mechanosignaling pathways. An oscillatory flow showed greater similarity in the translocation of MRTF-A and YAP1 and lower similarity in the translocation of β-catenin and RelA to the cells on the RPM.

## 4. Discussion

Microgravity enables entirely new approaches to cell biology studies, including cancer research on a cellular level (reviewed in [5,30,31]). It is well established among gravitational researchers that it is impossible to simulate an exact microgravity environment in the laboratory. Nevertheless, depending on the device and the cell system chosen, ground-based facilities can produce results that closely resemble those of space experiments [32,33,34]. Therefore, simulated microgravity bioreactors may be a useful tool for space scientists. However, devices like the RPM have been a biological “black box”, and most of the molecular and cellular processes that lead to exciting discoveries remain uncharacterized. In this study, we aimed to shed light on the cellular processes involved in cultivating adherent cancer cells on the RPM and understand how and why tumor spheroids form during culture in random positioning conditions.

### 4.1. Adherent Cells in Rotating Bioreactors

Investigation of gravitational biology is the most common reason for using rotating bioreactors. This requires that cells perceive changes in the direction of the gravity vector. While this property has already been proven for larger unicellular organisms (such as *Paramecium* or *Euglena*) [35,36,37], there has not been a clear answer for smaller human cells. Early calculations by Albrecht-Bühler suggest that the volume within a human cell is too small for the cell to sense gravitational changes [10]. Albrecht-Bühler instead assumed that the mass of the surrounding cell culture medium, which is also subject to gravity, triggers an indirect gravitational perception by exerting external pressure on the cell.

Restructuring of the cytoskeleton is often suggested as an early response to changes in the gravity vector [38]. Typical rearrangements of the cytoskeleton (reduced stress fibers, the formation of lamellipodia, etc.) were also observed by Ju et al. in their comparative study with different human cell types in response to simulated microgravity on a 3D clinostat. However, they observed the same cytoskeletal changes in an inverted cell culture, which they concluded must be a general response of mammalian cells to gravitational changes [39]. These effects could also be explained by the large column of culture medium above the cells in a standard orientated culture flask, which is not present in an inverted flask. However, it should be noted that the liquid column itself causes a hydrostatic pressure difference of 2.4 mbar in a 2.4 cm thick (*p* = *ρ* × *a* × *h*) completely filled T25 cell culture flask, which is in the order of magnitude of daily air pressure fluctuations. A possible (and maybe additive) influence of air pressure on cell culture observations should be investigated in a further study. Nevertheless, these results could explain the relaxation of tension the cell sensed, which may have induced the cytoskeletal changes observed. This could lead to the assumption that human cells in in vitro culture might sense only the indirect effects of gravity. In the meantime, however, other studies have appeared indicating that at least some human cells might be able to perceive gravity directly. Luna et al. [40] exposed human mesenchymal stem cells to different angular velocities on a clinostat and observed a rounder, less spread morphology as a function of the rotation speed. This angular frequency dependence suggested that the ability of a human cell to perceive the changing gravity vector depends on the rate of perturbation [40]. It is important to note that a slide, not a T25 culture flask, was used in this experiment to avoid the possible effects of shear forces and movement of the cell culture medium.

Clinorotation is usually associated with lower shear forces, which should result in a lower F-actin density in clinorotated cells compared with cells on the RPM. This was not observed in our experiments (Figure 2H); however, our goal was to study RPM motion and not to generate perfect clinorotation. One explanation for this discrepancy could be the use of adherent cells instead of suspension cells. In addition, the rotation axis of an RPM operated in clinostat mode is much more offset compared with a slideflask/channel slide clinostat, resulting in low centrifugal forces (about 0.01 *g* on the cell layer, assuming a 10 cm offset and a rotation speed of 60°/s).

Even assuming that human cells are gravisensitive and can sense gravity directly, the question remains as to which subcellular structures are responsible. Vorselen’s theory of the cytoskeleton as one of the first gravity responders in non-specialized cells [41] is still relevant, but is weakened by the fact that structural changes in the cytoskeleton happen in response to any mechanical influence on the cell, even with indirect perception of gravity. This is particularly evident as we observed changes in F-actin not only in response to the spatial orientation of the cell culture but also in response to the fluid flow without changing the gravitational vector (Figure 2). Also, the fact that MRTF-A and YAP1 are predominantly nuclear-localized in the rotating and flow cell cultures suggests that RhoA-dependent active actin remodeling occurred during fluid shear (Figure 2 and Figure 6).

Zhang et al. [42] also compared different orientations of cell culture flasks for investigating the effect of the gravity vector on the mechanical remodeling of adherent murine MC3T3-E1 preosteoblast cells. Their results proposed a biomechanical model for integrating the mechanosensation of nucleus displacement with cytoskeletal remodeling and reorganization of the focal adhesion complex triggered by the gravity vector. If the mechanical stability of orientation-varied adherent cells could be explained, among other things, by the sedimentation of cell nuclei, this finding would suggest that gravisensing mechanisms in plant cells (sedimentation of statoliths) could also apply to mammalian cells in a distinct way. These results could explain why adherent cells adapt to a new physical culture condition after a period of time, which we saw in FTC-133 when comparing the results after 4 and 72 h (Figure 1). While we detected a significant translocation of p38 into the cytoplasm in RPM-exposed cells after 4 h, this was no longer the case after 72 h (Figure 2).

### 4.2. Flow Shear

Previous studies with thyroid cancer cells on the RPM show that the first spheroids form between 12 and 24 h. Our experiments have shown that FTC-133 cells begin to detach after about 12 h in a flow channel, which should have a similar fluid flow to a culture flask on the RPM. This process would be consistent with the timings observed on the RPM. We have also seen that the spheroid formation of FTC-133 cells depends on the RPM rotational speed, which should be proportional to the expected shear forces. Interestingly, by doubling the flow rate in the flow channel, we did not observe earlier detachment of the cells. Only in the presence of air bubbles did the cells’ detachment occur from the beginning of the experiment.

We operated the RPM at different speeds to determine how shear stress affects biological experiments. After 8 h, the time-averaged gravity vector in all experimental setups was around 0.01 *g*, implying a “microgravity” condition. However, what distinguishes each setup from the others are the shear forces generated, which have already been calculated by Wuest et al. [14] for cell culture flasks on the RPM. Since the spheroid formation depended on the rotation speed, the microgravity simulation alone cannot be responsible for this process. When placed in a T25 flask at an average speed of 60°/s (commonly used in RPM microgravity simulation experiments), an estimated 70% of adherent cells experience a shear stress of at least 10 mPa. Further, 20% of the cells experience even higher rates of 25 mPa or more [14]. We mimicked these shear forces in a flow channel for ease of study and obtained a similar gene signature response to RPM experiments of the same length after 4 h.

Not all cells are exposed to the same shear forces on the RPM, which may have resulted in the lower fold changes in gene expression in the RPM-exposed cells compared to the cells from the flow channel. According to the simulations by Wuest et al. [14], only 20% of the cell monolayer was exposed to forces of the same magnitude as in the channel slide. There were a few differences in the nuclear levels of the transcription factors p38, MRTF-A, and β-catenin, suggesting that the mechanosignaling of cells on the RPM cannot be replicated using a simple flow experiment. An oscillatory flow mimicked the changing shear stress direction found on the RPM better than a unidirectional flow but could not explain the difference. This further illustrates that the detachment of adherent cells, essential for spheroid formation on the RPM, can be mimicked by the shear forces from fluid flows and suggests that adherent cell layers on the RPM are subjected to higher mechanical stress before spheroids form. This is also supported by the fact that genes overexpressed in the adherent RPM cell population were upregulated in cancer cells exposed to shear stress (Figure 6F). Recently, advances in the study of the effects of low shear stress on cancer cells have been published [43,44,45]. Of particular interest for random positioning research are the low shear stress rates of 10–25 mPa that are expected during normal RPM operation. The physiological shear values of the lymphatic system (10–20 mPa), for example, can be found in this range [43], which is of critical importance for experiments with metastatic cancer cells or those isolated from lymphatic system metastases such as FTC-133. Work by Lee et al. [44] showed that low shear stress can activate YAP-dependent motility programs in metastatic cancer cell lines. In 2022, Kim et al. [45] showed that the cell motility in prostate cancer cell lines depends on the Piezo1–Src–YAP axis at low shear stress.

Triggered by the fluid flow inside the flow channel, we observed that the cells detach, migrate, and roll across the surface in the same direction as the media flow. At some point, the cells rounded out, detached, and were carried away by the flow. This observation over an extended period suggests that the increased detachment of the cells induced by flow is a biological process. Cells can sense flow-induced shear stress depending on its intensity and direction in a YAP-dependent manner that promotes cell motility [44]. We confirmed that the shear forces also contribute to the increased proliferation in RPM cell cultures [46,47]. We observed that cell confluency increased more rapidly at a higher flow rate of 1 mL/min compared with 0.5 mL/min until approximately 12 h after the start of the experiment.

Numerous research groups have reported alterations in gene and protein expression related to focal adhesion components, including integrins, as well as changes in the activity of focal adhesion kinase components, when employing rotating bioreactors to simulate microgravity [41]. These changes may be attributable to the cellular motility within this environment and can be more appropriately understood as a general cellular response to low shear stress.

### 4.3. Air Bubbles

First of all, it must be said that most studies pay enough attention to remove air bubbles in their experiments in order not to generate misleading results. It is therefore unusual to introduce additional air bubbles into a culture vessel. The bubble experiment (Figure 5) was primarily designed to investigate the effects of relatively large air bubbles on the cells. Despite particular care during filling, air bubbles in culture flasks on the RPM cannot be completely avoided, especially during longer experiments of ≥24 h. We have investigated a number of possible causes for the occurrence of bubbles in RPM experiments (compare Figure S1). However, it is most likely a combination of several effects. The correlation between the occurrence of air bubbles and the formation of spheroids, which we have seen in many RPM experiments, could be supported by the flow channel experiments in this study (Figure 5). The movement of air bubbles over the cell layer resulted in the rapid detachment of cells. This helps produce a larger amount of spheroids but results in much greater mechanical stress on the adherent cell population. Air bubbles also cause significant problems in microfluidic devices, and it is usually recommended to avoid them [48]. Bubbles in motion can have different effects depending on their size. They can interact directly with the cells and turbulent flow can be generated behind rising bubbles. Bubbles’ effects on cell damage have been discussed for suspended cell populations. Cells can become trapped in the circulating fluid behind a rising bubble (“bubble wake”) and are thereby dragged along by the bubble. For small bubbles, these microeddies can be sufficiently intense to even create cell damage [49]. However, as soon as bubbles have formed in a culture flask on the RPM, they immediately hit the flask wall due to buoyancy and travel with rather high velocities along the wall with each revolution. These bubbles create high velocity gradients, and thereby high shear forces, in the liquid film between the bubble and cells [50].

### 4.4. Cell Density

Another interesting observation in our study was the influence of the cell pre-incubation time on RPM-induced spheroid formation. A longer pre-cultivation prior to RPM start resulted in a reduced formation to an absence of formation of 3D aggregates. It has previously been described that overgrown cell cultures no longer form spheroids under microgravity conditions [26]. In the case of a very dense cell culture, one possible explanation could be that there is no lateral surface of attack for shear forces due to the lack of space between the cells. Another explanation could be that cells detach during mitosis when they are less attached to the substrate (rounding of the cell). At low density, cells continue to divide and are at higher risk of detaching. At high density, the cells may go into a senescent state and stop dividing. Thus, the risk of detachment is lower. The results of the inverted flask experiments suggest that FTC-133 cells regularly detach from the surface, which would not be noticeable in a normally positioned cell culture (Figure 2). In cancer cells, this property can contribute to the formation of new metastases. However, further investigations are required to determine whether prior cell migration, cell–cell or cell–matrix contact formation, or mitosis is necessary for detachment (Figure 4). Our observations suggest that this is the case, as the detached cells migrated in the flow channel before their final detachment. The free space between the cells allows them to migrate according to the YAP nuclear status (Figure 2). YAP signaling is involved in contact inhibition of cell proliferation and growth [51] and allows the cell to maintain a motile environment by limiting the maturation of focal adhesions and restricting cytoskeletal tension [52]. Once a cell culture reaches confluence, YAP localization is completely cytoplasmic, and cells cease motility and possibly subsequent detachment in an RPM cell culture.

### 4.5. Spheroid Formation on the RPM

**Figure 8:**
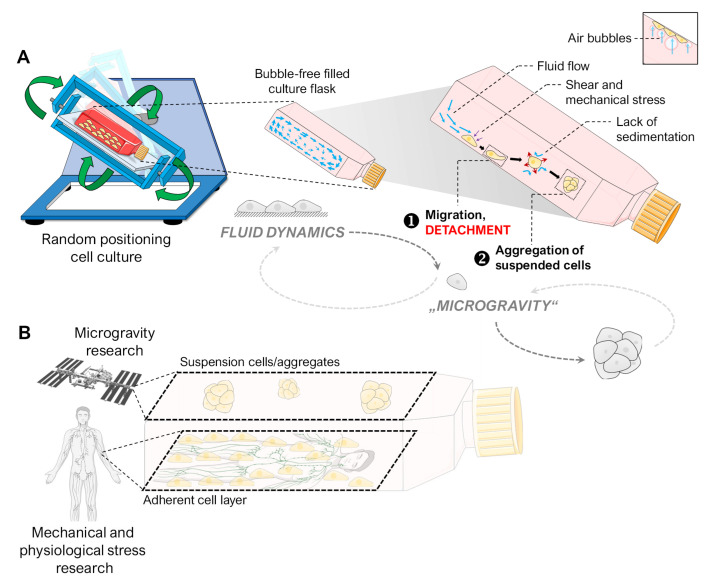
(**A**) Illustration of the hypothesized processes occurring with adherent cells in an initially air-bubble-free cell culture flask on the RPM, finally leading to spheroid formation. We observed that RPM-induced spheroid formation is a two-step process. First, the adherent cells detach due to mechanical stress (e.g., fluid flow, air bubbles). Then, “simulated microgravity” (free-fall) leads to the formation of three-dimensional spheroids by preventing the sedimentation of suspended cells. The small picture indicates the amplifying mechanical effect of air bubbles. (**B**) The two cell populations (adherent cell layer and suspension cells) of an RPM cell culture of adherent cells are subjected to different mechanical forces. While the suspension cells are held in a mostly stress-free suspension, the cell layer experiences shear forces similar to those in the human lymphatic system. Parts of the figure were drawn by using pictures from Biorender.com and from Servier Medical Art.

Our experiments indicated that the spheroid formation of adherent cells on the RPM is likely to be a two-step process (Figure 8A). In the first step, the cells are detached from their monolayer. Responsible for this are mainly fluid shear and the mechanical forces of migrating air bubbles during random positioning. Our fluid channel experiment mimicked fluid flow at a comparable scale to the RPM regular operation [14] and induced similar translocation of mechanosensitive transcription factors and a comparable signature of activated mechanoresponsive genes in FTC-133 cells. After detachment, the cells on the RPM are suspended in free-fall due to the appropriate rotational speed, preventing their sedimentation and two-dimensional outgrowth. In this phase, the cells aggregate into three-dimensional aggregates. The work of Melnik et al. [18] has shown that FTC-133 cells, when used as a suspension on the RPM, also form spheroids. Therefore, starting an RPM experiment with a layer of adherent cells is not essential for generating and studying spheroids. In other devices for microgravity research, such as the ClinoStar (CelVivo, Denmark) or the rotating wall vessel bioreactor (Synthecon, TX, USA), cells are also seeded in suspension before floating spheroids develop under rotation. However, using adherent cells in an RPM experiment allows for additional studies. For example, not all cells detach equally due to random positioning. A balance of adhesion and anti-adhesion proteins was discussed as responsible for this behavior [18]. In addition, metastatic cancer cells tolerate the suspension phase better due to the anoikis (cell death due to loss of cell–matrix contact) resistance they usually develop during the metastasis formation multistep process [53], as a prolonged culture phase before the start of random positioning inhibited the detachment of FTC-133 cells (Figure 3). After a two-day pre-attachment phase, the only conditions that obtained spheroids were higher angular velocities, which were only able to induce the formation of a single small spheroid (ca. 110 µm) (Figure 3). The cells exhibited no transcriptional response to RPM treatment until higher velocities were applied. However, whether this inertia was related to increased “stickiness” due to a plentiful secreted extracellular matrix, to a higher level of adhesion proteins, or simply to the physical protection against flow shear caused by being embedded into a cell layer cannot be deduced from our data.

### 4.6. Implications for Future Experiments in Gravitational Biology

#### 4.6.1. “Simulated Microgravity”

The RPM continues to be a suitable bioreactor to simulate microgravity effects for some cell model systems on Earth. To achieve optimal results in cell culture experiments, it is vital to have a thorough understanding of the internal dynamics of random positioning. Attention to these dynamics ensures that experiments are well planned and yield accurate and reliable results. For a suspended object, the RPM can simulate free-fall, the main effect experienced by astronauts and organisms on the ISS or during parabolic flights. An adherent cell population on the RPM is subject to shear forces that detach the cells after some time, leaving them to float free in the culture medium. Without sedimentation, if they can resist anoikis, they may aggregate and form spheroids.

It must be considered that the adherent cell layer and the suspension cells are exposed to very different mechanical forces, especially when air bubbles are present. Meaningful results will only be achieved with careful design of RPM-based experiments. The cellular effect triggered by fluid motion can be several orders of magnitude higher than the effect triggered by gravity alteration. Thus, it is not necessary to design a gravity-focused RPM experiment with a prior adhesion period leading to a two-dimensional cell layer. Instead, initiation with suspension cultures could prove a possible alternative. In 2022, Masini et al. [54] successfully performed a long-term experiment with suspended cancer cells on the RPM. They found that under the influence of the RPM, the behavior and metabolism of tumor cells led to the acquisition of an aggressive and metastatic stem cell-like phenotype.

The size and geometry of the cell culture vessel also play a role, as they directly influence the turbulence of the medium generated during random positioning. In the current study, we also observed this effect when using different culture flasks for the FTC-133 cells. In large flasks, the medium has a higher rotational energy. In small channel slides, the adhesive forces dominate, and the medium has hardly any chance to reach its full speed. Lower shear stress in the slide flasks or channel slides resulted in fewer or no spheroids, respectively, even though the RPM program or random speed and random direction velocities used were the same.

#### 4.6.2. Real Microgravity

Spheroid formation of adherently growing human cells was also observed in several space experiments. In the ISS experiments SimBox [1], SPHEROIDS [2], and CellBox-2 [3], the formation of 3D aggregates in real microgravity has been reported. Since the detachment of cells has not yet been observed in microgravity, it cannot be completely ruled out that cells detach from their substrate during the microgravity phase. Nevertheless, it is possible that the appearance of these spheroids was triggered by a specific orientation of the flight hardware (e.g., by flipping) in combination with the formation of air bubbles (compare Figure S1). In our study, we were able to show that an upside-down-orientated cell culture flask with air bubbles led to the formation of spheroids after some time (Figure 2). Since it is unknown and not determinable in which orientation the flight hardware was stored between the handover and the rocket launch, a similar effect in the CellBox-2 experiment cannot be excluded. Accordingly, spheroids could have formed even before arrival on the ISS, e.g., due to accelerations or vibrations during the launch phase. FTC-133 cells were also used in the space experiment, and numerous air bubbles could be seen in the culture chamber at the end of the experiment (Figure S1B). Live cell observation during space experiments would help to determine when spheroids are formed during spaceflights. In future experiments on spheroid formation in real microgravity, special care must be taken to avoid air bubbles, e.g., using a bubble trap in the loop system or specific culture flask geometries that minimize the described bubble effect (e.g., channel or chamber slides).

#### 4.6.3. Cell Models

While individual suspension cells are directly exposed to direct or indirect gravitational effects on the RPM, this is not necessarily true for cells inside cell clusters or multicellular organisms. While the cells of multicellular plants with statoliths still have intracellular perception mechanisms for gravity [55], mammalian cells have established perception of external structures exposed to gravity (e.g., otoliths). The cells used for the gravitational perception of environmental stimuli are mostly located on the internal or external surfaces of the organism, sometimes combined into sensory tissues or organs. The task of perceiving gravity is delegated to a higher organizational level (organ or system). Cells embedded into mostly non-gravity-sensitive tissue are likely to respond only to the mechanical deformation of the surrounding tissue but not to changes in the gravity vector. This may also be true for position-fixed adherent cells in a monolayer on the RPM. In 2022, ElGindi et al. [56] provided evidence of the more influential role of the local microenvironment in comparison to the general gravity vector. Their experiments showed that a 3D microenvironment attenuated the changes in the transcriptome of T cells mediated by the RPM. The research group also exposed dendritic cells to the RPM in two different types of extracellular matrices (loose and stiff) [57]. The effects of random positioning at the transcriptome level were smaller when the cells were cultured in the denser matrix. Both studies indicate the major influence of the microenvironment on mammalian cell biology. This is also supported by the different effects at the transcriptional and protein levels found in numerous comparative analyses of the two cell populations (adherent cells, AD, and multicellular spheroids, MCS) in RPM experiments with human cells [8,19,46,58]; although both populations are by definition subjected to “simulated microgravity”, the adherent cell layer is more exposed to mechanical fluid flow, whereas the spheroid cells float in a suspension with lower shear and are also primarily embedded into a protective stable tissue aggregate. Here, the cell microenvironment probably has a greater influence than the constantly changing gravitational vector and can influence cell signaling, proliferation, and apoptosis [59,60,61]. One possible approach to answering this ambiguous question is the planned project by Larose and colleagues to study spheroids and organoids under real microgravity conditions. This will open up the possibility of testing the extent to which microgravity affects the three-dimensional constructs of cancer cells [62].

Albrecht-Bühler [10] once commented that the microgravity simulation requires more than averaging the gravity vector in clinorotation. In addition, Einstein [63] stated that: “The gravitational field has only a relative existence in a way similar to the electric field generated by magnetoelectric induction. Because for an observer falling freely from the roof of a house there exists—at least in his immediate surroundings—no gravitational field”. After more than 100 years, we can confirm this at least for human cells. While probably only isolated cells free-falling in vacuum could experience microgravity directly (a theoretical consideration), the indirect effects of altered gravity are perceived and processed differently by cells, depending on their type, origin, and cell culture conditions, and the characteristics of the “microgravity simulator” used. Conversely, this also means that a free-fall simulator is not always required to mimic the cellular effects of exposure to microgravity. A cell type-specific combination of mechanical stimuli could be sufficient.

### 4.7. Study Limitations

It should be noted that we have a heterogeneous cell population on the RPM (because not all cells are subjected to the same shear forces). Since we saw that not all cells detached simultaneously during flow exposure, we can hypothesize that cells may regulate some molecular factors before detaching from the surface. Unfortunately, we could only perform endpoint analyses from the localization of transcription factors and gene expression. Live cell observation of the cells on the RPM would provide more accurate information on spontaneous and/or short-term changes and adaptations. Some researchers suspect that the vibrations inside an incubator prevent/slow cell attachment to plastic flask surfaces. In the current study, we did not investigate the influence of vibrations, but started the experiments after a preceding 24(-48) h attachment phase of the cells.

## 5. Conclusions

Spheroid formation of adherently growing cells on the RPM is a two-step process. It requires detachment of the cells, caused mainly by fluid flow (triggered by the rotation of the cell culture flask), and air bubbles can enhance it. Once the cells are in suspension, random positioning prevents sedimentation, and 3D aggregate formation occurs.

The detachment itself seems to depend on the balance between the cell-specific adhesion forces (depending on the cell density, amount of matrix, and adhesion proteins) and the physical shear forces of the cell culture (flow rate, bubbles, surface conditions of the cell culture flask). In the current study, a fluid flow experiment mimicked several RPM-induced effects in adherent FTC-133 cells. During regular operation of the RPM, shear forces act in a range similar to that of the human lymphatic system. Thus, the RPM serves as a free-fall simulator for (detached) suspension cells in gravitational research, but it also simulates the shear forces of the human organism on the adherent cell layer, providing a unique combination of study conditions for cancer research (Figure 8B). To ensure that, experiments with the RPM must be well planned and the results interpreted accordingly.

## Data Availability

The data presented in this study are available in the electronic Supplementary Materials. The complete raw data are available on request.

## Supplementary Materials

The following supporting information can be downloaded at https://www.mdpi.com/article/10.3390/cells12222665/s1: Figure S1: Occurrence of air bubbles under different cell culture conditions and handling of cell culture flasks; Table S1: Antibodies used for immunofluorescence analyses; Table S2: Primer sequences for quantitative real-time PCR; Video S1: 24 h time-lapse video of FTC-133 cells exposed to a fluid flow of 1 mL/min inside a channel slide; Video S2: 24 h time-lapse video of FTC-133 cells exposed to a fluid flow of 1 mL/min with 1 bubble/min inside a channel slide.
