# Supplementary material for: Fluid and Bubble Flow Detach Adherent Cancer Cells to Form Spheroids on a Random Positioning Machine
Source: Cells. 2023 Nov 20;12(22):2665. doi: 10.3390/cells12222665 (PMC10670461; doi:10.3390/cells12222665)
Supplement: Supplementary file 1 [file cells-12-02665-s001.zip › cells-2717437-supplementary.pdf]

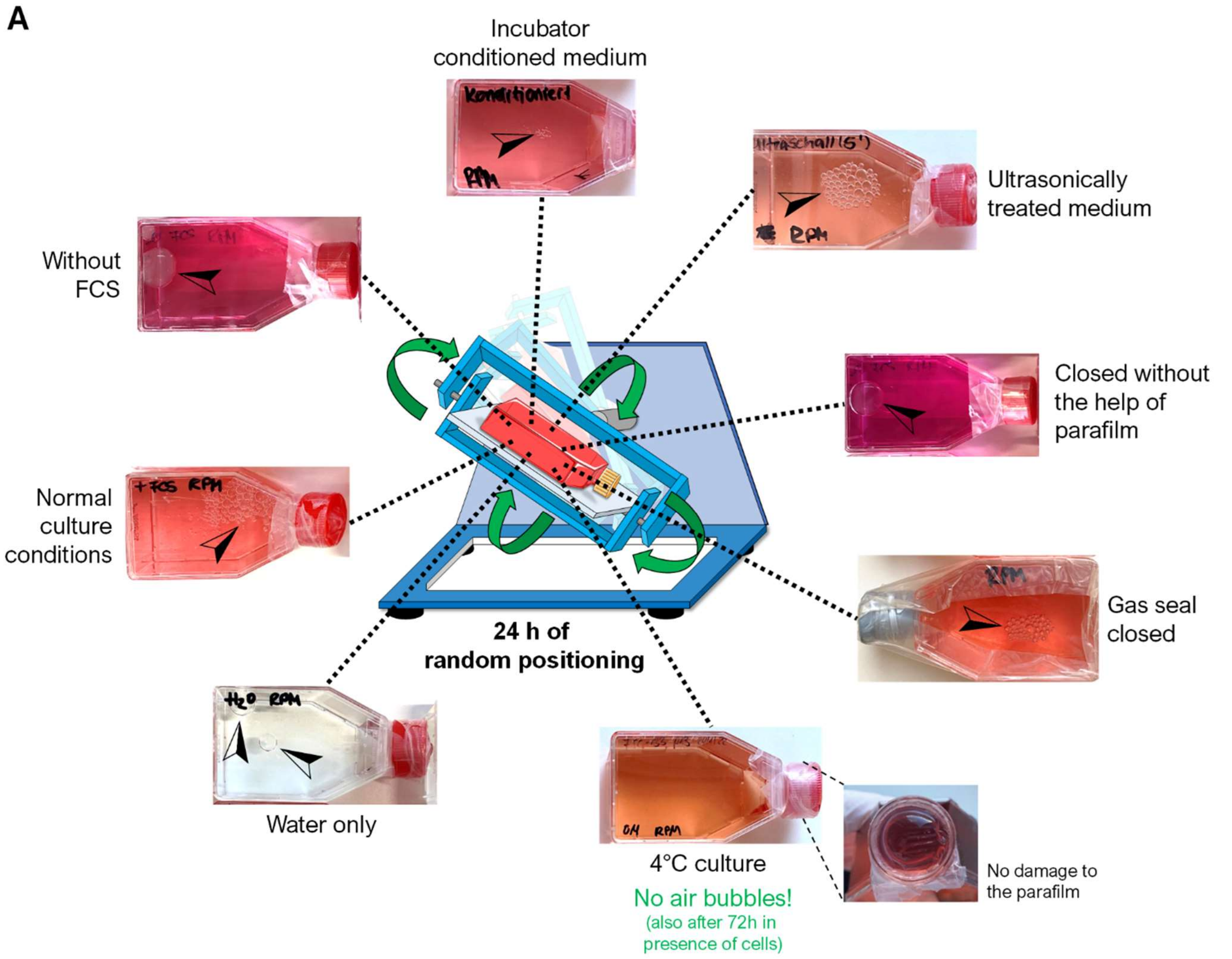

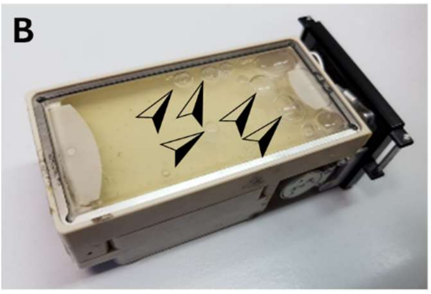

Figure S1. Occurrence of air bubbles under different cell culture conditions and handling of cell culture flasks. (**A**) Bubble formation (indicated by black arrows) in bubble-free filled T25 cell culture flasks after 24 h of random positioning under different conditions (without cells). (**B**) Flight container of the CellBox-2 experiment after its return from the ISS. Parts of the figure were drawn by using pictures from Servier Medical Art.

Table S1. Antibodies used for immunofluorescence analyses.

**Antibody** (anti-…)	**Company, Product Nr.**	**Species**	**Dilution IF**
β-catenin	Invitrogen, #71-2700	Rabbit	1:100
p38 MAPKα	Invitrogen, #MA5-15116	Mouse	1:1000
MRTF-A/MKL1	Santa-Cruz, #sc-390324	Rabbit	1:100
NFκB p65 (RelA)	Invitrogen, #510500	Mouse	1:500
YAP1	Sigma-Aldrich, #WH0010413M1	Mouse	1:1000

**Secondary antibody**	**Labelling**	**Company, Product Nr.**	**Dilution**
anti-Mouse	AF488	Invitrogen, #A1101	1:1000
	AF647	Invitrogen, #A21235	1:1000<br>for MRTF-A: 1:500
anti- Rabbit	AF488	Invitrogen, #A11008	1:1000
	AF647	Invitrogen, #A21244	1:500

AF, Alexa Fluor; IF, immunofluorescence.

Table S2. Primer sequences for quantitative real-time PCR.

Gene	Primer Name	Sequence (5′->3′)
18S rRNA	18S-F	ggAgCCTgCggCTTAATTT
	18S-R	CAACTAAgAACggCCATgCA
ANKRD1	ANKRD1-F	AgCgCCCgAgATAAgTTgC
	ANKRD1-R	gTCTgCCTCACAggCgATAA
CAV1	CAV1-F	CCACgggCCAgCATgT
	CAV1-R	gCCTTgTTgTTgggCTTgTAg
CCN2 (CTGF)	CTGF-F	ACAAgggCCTCTTCTgTgACTT
	CTGF-R	ggTACACCgTACCACCgAAgAT
CXCL8	IL8-F	TggCAgCCTTCCTgATTTCT
	IL8-R	gggTggAAAggTTTggAgTATg
FN1	FN1-F	AgATCTACCTgTACACCTTgAATgACA
	FN1-R	CATgATACCAgCAAggAATTgg
ICAM1	ICAM1-F	CggCTgACgTgTgCAgTAAT
	ICAM1-R	CTTCTgAgACCTCTggCTTCgT
IL6	IL6-F	CgggAACgAAAgAgAAgCTCTA
	IL6-R	gAgCAgCCCCAgggAgAA
KRT8	KRT8-F	gATCTCTgAgATgAACCggAACA
	KRT8-R	gCTCggCATCTgCAATgg
SNAI1	SNAI1-F	CCAgTgCCTCgACCACTATg
	SNAI1-R	CTgCTggAAggTAAACTCTggA
VCL	VCL-F	gTCCACCAgCCCTgTCATTT
	VCL-R	gTCTCggCTgCTCgTATCTT
VEGFA	VEGFA-F	CTACCTCCACCATgCCAAgTg
	VEGFA-R	gCgCTgATAgACATCCATgAAC
